# Increased Duodenal Iron Absorption through Upregulation of Ferroportin 1 due to the Decrement in Serum Hepcidin in Patients with Chronic Hepatitis C

**DOI:** 10.1155/2018/2154361

**Published:** 2018-08-14

**Authors:** Masanori Sato, Koji Miyanishi, Shingo Tanaka, Akira Sakurada, Hiroki Sakamoto, Yutaka Kawano, Kohichi Takada, Masayoshi Kobune, Junji Kato

**Affiliations:** ^1^Department of Medical Oncology, Sapporo Medical University School of Medicine, South-1, West-16, Chuo-Ku, Sapporo 060-8543, Japan; ^2^Department of Medical Hematology, Sapporo Medical University School of Medicine, South-1, West-16, Chuo-Ku, Sapporo 060-8543, Japan

## Abstract

Hepatic iron accumulation is generally increased in the chronic hepatitis C (CHC) liver; however, the precise mechanism of such accumulation remains unclear. We evaluated iron absorption from the gastrointestinal tract of patients with CHC and control participants. We measured the expression of a panel of molecules associated with duodenal iron absorption and serum hepcidin levels to determine the mechanism of iron accumulation in the CHC liver. We enrolled 24 patients with CHC and 9 patients with chronic gastritis without* Helicobacter pylori* infection or an iron metabolism disorder as control participants. An oral iron absorption test (OIAT) was administered which involved a dosage of 100 mg of sodium ferrous citrate. Serum level of hepcidin-25 was measured by liquid chromatography-tandem mass spectrometry. Ferroportin 1 (FPN) mRNA was measured by RT-PCR and FPN protein was analyzed by western blot. Samples were obtained from duodenum biopsy tissue from each CHC patient and control participant. Caco-2/TC7 cells were incubated in Costar transwells (0.4 *μ*m pores). The OIAT showed significantly greater iron absorption in CHC patients than control participants. Serum hepcidin-25 in the CHC group was significantly lower than in the control group. Compared with control participants, duodenal FPN mRNA expression in CHC patients was significantly upregulated. The FPN mRNA levels and protein levels increased significantly in Caco-2/TC7 cell monolayers cultured in transwells with hepcidin. Lower serum hepcidin-25 levels might upregulate not only FPN protein expression but also mRNA expression in the duodenum and cause iron accumulation in patients with CHC.

## 1. Introduction

Hepatic iron overload has long been known to develop in patients with chronic hepatitis C (CHC) [1]. We previously revealed that excess free iron in hepatocytes promotes not only the production of reactive oxygen species (ROS) causing damage to hepatocytes and hepatic fibrosis but also increases the level of 8-hydroxy-2′-deoxyguanosine (8-OH-dG), a mutagenic base, in the liver, resulting in the development of hepatocellular carcinoma (HCC). In addition, we found that iron reduction therapy in combination with phlebotomy and a low iron diet improved hepatitis and hepatic fibrosis while normalizing the elevated 8-OH-dG level [2]. We also found that long-term iron depletion significantly reduced the incidence of HCC in patients with CHC (F2/F3 grade) [3]. However, the precise mechanisms involved in iron overload remain to be clarified.

The body's iron supply is obtained largely by iron absorption from the intestine and reabsorption from hemoglobin in the reticuloendothelial system [4]. Intestinal iron absorption is mainly from the duodenum. Two iron transport proteins, divalent metal transporter 1 (DMT1) and ferroportin 1 (FPN), are expressed in duodenal enterocytes [4, 5]. Hepcidin is a 2.7 kDa peptide consisting of 25 amino acids that is produced mainly in hepatocytes and acts as a major regulator of iron homeostasis [6]. Hepcidin affects macrophages, Hela cells, or human embryonic kidney cells, so that they regulate iron efflux and storage and the duodenum so that it inhibits iron absorption [7–9]. Although several reports have shown decreased expression of hepcidin in CHC patients [10, 11], no reported study has investigated the dynamics of iron absorption in patients with nonalcoholic steatohepatitis.

In the current study, we aimed to investigate the mechanism of hepatic iron overload in CHC patients. For this purpose we measured the expression of a panel of molecules in association with iron absorption in the duodenum and serum hepcidin levels.

## 2. Materials and Methods

### 2.1. Patients and Tissue Samples

Duodenum tissue samples were obtained from 24 CHC cases between February 2008 and January 2016. In addition, biopsied duodenum tissue samples were examined from 9 control participants with chronic gastritis who did not have* Helicobacter pylori* infection or an iron metabolism disorder. The ethical committee for human genome/gene analysis research of our hospital approved this study. All participants provided written informed consent at the time of tissue sampling to take part in this study after receiving a detailed explanation of the procedures involved according to the Declaration of Helsinki.

### 2.2. Criteria for Definition of CHC

CHC was defined according to an ALT value ≥30 U/L and HCV RNA positivity by polymerase chain reaction (PCR). Patients younger than 18 years and those with hepatitis B surface antigen (HBs Ag) positivity, previous IFN treatment, serum HCV RNA negativity, and history of heavy alcohol abuse were excluded from this study.

### 2.3. Laboratory Tests

Laboratory tests were done at the same time as tissue sampling. Venous blood samples were taken in the morning after a 12-h overnight fast. Complete blood cell counts, determinations of serum iron, ferritin, total iron-binding capacity, and %transferrin saturation (Tf sat), and biochemical examinations, including that for serum ALT, were performed using automated procedures in the clinical laboratories of Sapporo Medical University Hospital (Sapporo, Japan).

### 2.4. Oral Iron Absorption Test

Iron absorption from the gastrointestinal (GI) tract was measured using the oral iron absorption test (OIAT) established in our laboratory as described previously [12, 13]. In brief, at 9 a.m. after an overnight fast a blood sample was taken to measure serum iron and total iron-binding capacity (TIBC). Subsequently, the participant ingested 100 mg of iron in the form of two tablets of sodium ferrous citrate (SFC). Blood samples for measurement of iron parameters were taken after 15, 30, 60, and 120 min. Participants were not permitted to eat during the test.

### 2.5. Serum Hepcidin-25 Concentration

Serum concentration of hepcidin-25 was measured by liquid chromatography-tandem mass spectrometry (LC-MS/MS) as described previously [13, 14]. The assay was performed by Medical Care Proteomics Biotechnology Co., Ltd. (Kanazawa, Japan).

### 2.6. Quantitative Real-Time Reverse-Transcriptase Polymerase Chain Reaction

We followed the methods of our previous report [13]. Assay numbers for the endogenous control (18S ribosomal RNA [rRNA]) and target genes were as follows: Hs00167206 m1 (DMT1) and Hs00205888 m1 (FPN). Total RNAs were extracted from cultured cells using RNeasy Mini Kits (QIAGEN, Valecia, CA, USA) according to the manufacturer's protocol. cDNA was prepared using the Super Script VILO kit (Invitrogen, Carlsbad, CA, USA). The cDNA was added to the TaqMan Universal PCR Master Mix (Applied Biosystems, Tokyo, Japan). Each mixture was transferred to a 96-well optical tray. Target mRNAs were reverse transcribed at 60°C for 30 min, followed by a PCR cycle with a melting step for 20 s at 94°C and annealing for 1 min at 60°C for a total of 40 cycles using the ABI PRISM 7300 Sequence Detection System (Applied Biosystems). The parameter Ct was defined as the fractional cycle number at which the fluorescence generated by cleavage of the probe passes a fixed threshold above baseline. The standard curve was constructed with 10-fold serial dilutions of total RNA from Caco-2/TC7 cell monolayers (calibrator) and composed of 4 points (200, 20, 2, and 0.2 ng of total RNA). The target RNA and relative messages to the calibrator, termed [Target]c and [18S rRNA] c, respectively, in unknown samples were quantified by measuring Ct and using a standard curve to determine the starting target message quantity. The final relative mRNA expression level was expressed as follows: relative target mRNA level = [Target] c/[18S rRNA] c. We performed duplicate measurements for each data point. Each RT-PCR run included 5 points on the standard curve, a no-template control, the calibrator total RNA, and 36 unknown total RNAs. All of the samples with a coefficient of variation for relative messages to the calibrator data >10% were retested as previously reported [13, 15].

### 2.7. Western Blot Experiments

Proteins were extracted from cultured Caco-2/TC7 cell monolayers using RIPA buffer (150 mmol/L NaCl, 1% Nonidet P-40, 0.5% deoxycholate, 0.1% sodium dodecyl sulfate, 50 mmol/L Tris HCl pH 8.0, 0.2 mmol/L phenylmethylsulfonyl fluoride, 1 *µ*g/mL pepstatin, 0.5 *μ*g/mL leupeptin), and 50 *μ*g of total protein was used for immunoblotting. Blots were incubated with 1 *μ*g/mL anti-DMT1 antibody or FPN antibody for 24 h at 4°C. For western blots, the specific antibodies used were: DMT1 catalog #ab55735 from abcam Cambridge, MA; and FPN #LS-B1836 from Lifespan Bioscience, Seattle, WA.

### 2.8. Cell Cultures

Caco-2/TC7 cells were obtained from American Type Culture Collection (ATCC; Manassas, VA, USA). To achieve a fully differentiated monolayer, cells were grown in Dulbecco's modified Eagle's medium (DMEM) containing 10% fetal bovine serum (FBS) on Costar transwell membrane inserts with 0.4 *μ*m pores (Corning, St. Louis, MO, USA) for 21 days. At the 22nd day of culture, serum-free DMEM was added to the upper well and DMEM containing 2% FBS was added to the lower well after which the cells were cultured for 48 h. At the 24th day of culture, DMEM containing 2% FBS was added to the upper well and the cells were treated with DMEM containing 10% FBS either with or without human 25-amino acid synthetic hepcidin for 48 h. We followed the methods of our previous report [13].

### 2.9. Immunohistochemistry

Biopsy tissues were fixed in 10% buffered formaldehyde. After overnight fixation, they were embedded in paraffin wax and 4-mm thick sections were cut. All immunohistochemical staining was carried out on a Leica Bond-Max autostainer (Leica Biosystems, Nussloch, Gmbh, Germany) according to the manufacturer's protocol, with appropriate positive and negative controls. Sections were baked at 60°C for 60 min in a dehydration oven, then dewaxed in Bond Dewax solution, and rehydrated in Bond Wash solution (Leica Microsystems). Antigen retrieval was carried out at pH 6 with Novocastra Epitope Retrieval solution (Leica Bioosystems) for 20 min at 100°C. Slides were then incubated for 15 min at room temperature with ferroportin antibody (HPA065634 dilution 1 : 150; Atlas Antibodies Stockholm, Sweden).

The staining intensity of FPN in the duodenum biopsy tissues was evaluated by the immunohistochemical score. The method was described previously [16, 17].

### 2.10. Statistical Analysis

Statistical evaluation was performed with GraphPad Prism (GraphPad Inc, La Jolla, CA, USA). Quantitative values are expressed as means ± standard deviation (SD) or median (range). Each data set was first evaluated for normality of distribution by the Kolmogorov-Smirnov's test to decide whether a nonparametric rank-based analysis or a parametric analysis should be used. Two groups were compared using Fisher's exact test for categorical data and either the Wilcoxon-Mann-Whitney U test or the one-way analysis of variance (ANOVA) for quantitative data. Spearman's rank correlation was used to quantify the association between continuous or ordered categorical variables. Comparison of chronologic changes in parameters in the OIAT was done by repeated-measures ANOVA. Comparison of changes in the starting target message quantity in qRT-PCR was made using the Jonckheere-Terpstra test.

## 3. Results

### 3.1. Characteristics of the CHC Group and Control Group

The levels of AST, ALT, serum iron, serum ferritin, and TF sat in the CHC group at diagnosis were significantly higher than those in the control group. There were no between-group differences in gender or hemoglobin, body mass index, and total cholesterol and triglyceride levels (Table 1).

### 3.2. Oral Iron Absorption Test

**Figure 1:**
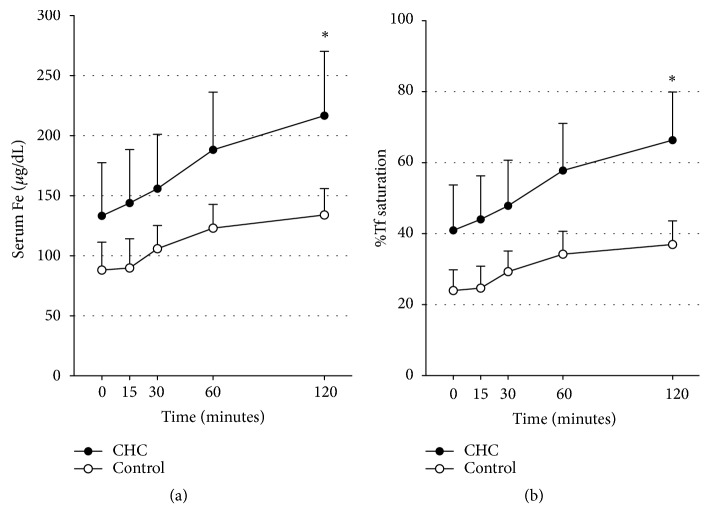
Serial changes in sFe (a) and Tf sat (b) after administration of sodium ferrous citrate. Closed circles indicate patients with CHC; open circles indicate controls. Compared to the control group, sFe and Tf sat were significantly increased in the patients with CHC (repeated-measures ANOVA. ^*∗*^*P* <0.001). These data show that iron absorption from the GI tract was significantly increased in the CHC patients.

To investigate the mechanism of hepatic iron overload in CHC patients, we performed the OIAT to evaluate iron absorption from the intestine. Compared with the control group, iron absorption was significantly increased from the GI tract in the CHC group (Figure 1(a);* P* < 0.001). Also, Tf sat was significantly increased in the CHC group compared with the control group (Figure 1(b);* P* < 0.001). Despite the increased Tf sat levels before the administration of SFC, iron absorption was upregulated in CHC patients.

### 3.3. Serum Hepcidin-25 Concentrations

**Figure 2:**
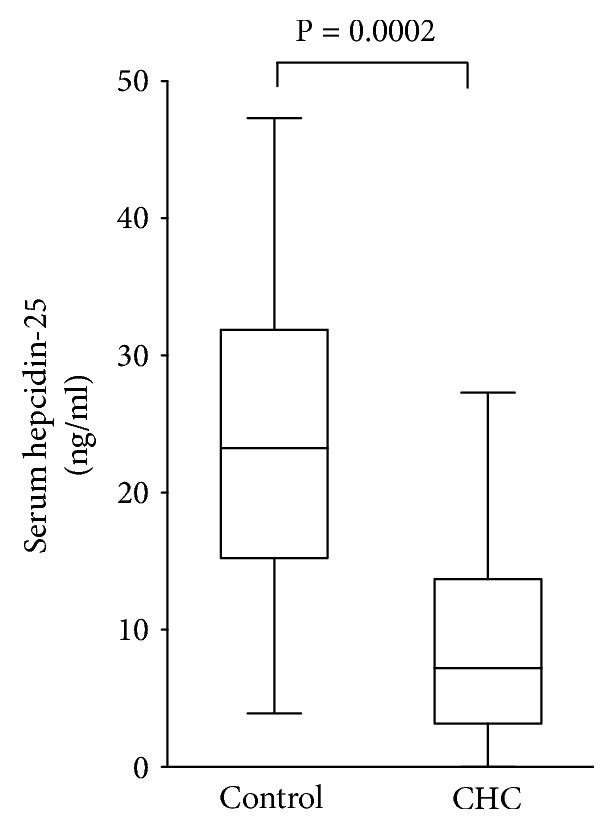
Serum hepcidin-25 levels were measured by LC-MS/MS. Median serum hepcidin-25 levels of control participants and CHC patients were 23.3 ng/mL (range, 3.9-47.3) and 7.2 ng/mL (range, 0-27.3). Serum hepcidin-25 levels in patients with CHC were significantly lower than in the control group (*P* = 0.0002; Wilcoxon-Mann-Whitney U test). Bottom and top edges of the boxes are the 25th and 75th percentiles, respectively. Median values are shown by a line within the box.

We measured serum hepcidin-25 using LC-MS/MS. The normal range of serum hepcidin-25 using LC-MS/MS analysis was reported to be 10-34 ng/mL [18]. The median serum hepcidin-25 level in the CHC group was 7.2 ng/mL (range, 0-27.3) and was 23.3 ng/mL (range, 3.9-47.3) in the control group, with the CHC group having a significantly lower value (Figure 2; P = 0.0002). Serum  hepcidin-25 level was significantly correlated with OIAT s-Fe 120 min (*ρ* = -0.438 and p = 0.0196).

### 3.4. mRNA Expression of FPN and DMT1 in the Duodenum

**Figure 3:**
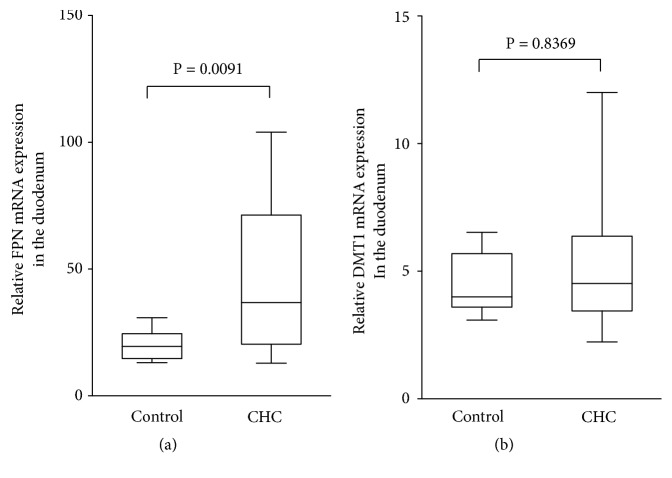
FPN and DMT1 mRNA expression in the duodenum. Relative mRNA expression levels of FPN (a) and DMT1 (b) in the duodenum were measured by Taqman real-time PCR. FPN mRNA in the CHC group was significantly upregulated compared with control participants (P = 0.0091; Wilcoxon-Mann-Whitney U test). Relative mRNA expression levels of DMT1 were measured by Taqman real-time PCR (b). There was no significant between-group difference (P = 0.8369; Wilcoxon-Mann-Whitney U test).

To investigate the mechanism of iron absorption from the intestine, we utilized Taqman real-time RT-PCR to evaluate the mRNA expression of molecules related to iron absorption in biopsy specimens from the duodenum. Median FPN mRNA level in patients with CHC was 36.8 (range, 12.9-104.0) and that in control participants was 19.5 (range, 13.1-30.8); values were significantly higher in CHC patients than in control participants (Figure 3(a); P = 0.0091). On the other hand, the median DMT1 mRNA level in the CHC group was 4.5 (range, 2.23-12.0) and 3.99 (range, 3.08-6.52) in the control group, with no significant between-group difference (Figure 3(b); P = 0.8369). These findings suggested that decreased serum hepcidin-25 levels and the resultant upregulated expression of FPN in the duodenum could promote iron absorption from the intestine, thereby causing hepatic iron overload in CHC patients.

### 3.5. Immunohistochemical Detection of FPN in Duodenum Biopsy Tissues

**Figure 4:**
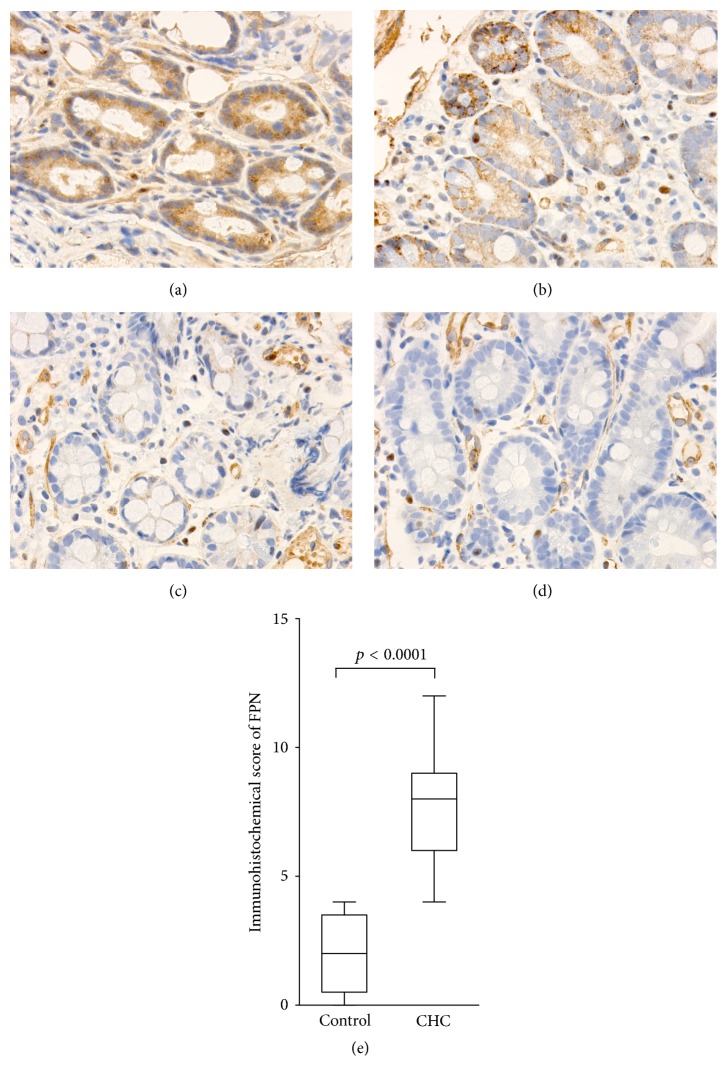
Immunohistochemical analysis of FPN in duodenum biopsy tissues from patients with CHC (a, b) and those of control participants (c, d). FPN expression was higher in the CHC group than in the control group. The immunohistochemical score was significantly higher in CHC patients compared with control participants (e).

To evaluate protein expression of FPN in the duodenum, we performed immunohistochemical detection in biopsy specimens from the duodenum using an anti-SLC40A1 antibody. In comparison with the control group, high FPN expression was observed in the duodenum specimens from CHC patients (Figures 4(a), 4(b), 4(c), and 4(d)). FPN expression was determined by scoring the intensity of immunohistochemical staining and comparing the results between the two groups. The immunohistochemical score for the CHC group was higher than for the control group (Figure 4(e); P < 0.0001).

Thus, these investigations using clinical specimens clearly showed increased iron absorption from the intestine in CHC patients compared with the control participants. These findings suggested that decreased serum hepcidin-25 values and increased FPN expression at both mRNA and protein levels could contribute to the mechanism of the increased iron absorption in CHC patients.

### 3.6. Induction of FPN in Caco-2/TC7 Cell Monolayers Cultured with Hepcidin

**Figure 5:**
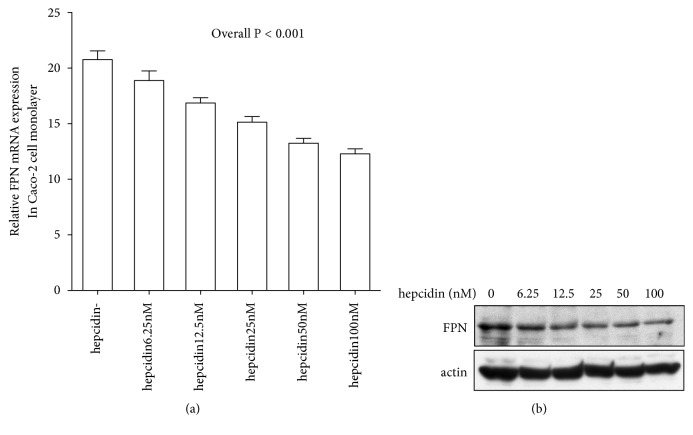
FPN expression in Caco-2/TC7 cell monolayers. FPN mRNA expression levels in Caco-2/TC7 cell monolayers cultured with or without hepcidin were measured by Taqman real-time PCR (a). FPN mRNA levels in Caco-2/TC7 cell monolayers were downregulated dependent on hepcidin concentration (P <0.0001; Jonckheere–Terpstra test). FPN protein expression was analyzed by western blot (b). FPN protein in Caco-2/TC7 cell monolayers was downregulated dependently on hepcidin concentration.

Although it was reported that hepcidin negatively regulates FPN at the protein level in reticuloendothelial cells [7], no study has directly addressed the interaction between hepcidin and FPN in the duodenal epithelium. We therefore examined the effects of hepcidin on the expression of two iron transport proteins* in vitro*. Caco-2/TC7 cells were cultured in a monolayer with or without hepcidin, and the difference in FPN and DMT1 expression was then investigated. The medium contained hepcidin at 5 different concentrations, from 6.25 to 100 nM. Relative FPN mRNA expression in Caco-2/TC7 cell monolayers was 20.46 (16.76-26.14) with no hepcidin, 18.40 (15.26-23.62) with hepcidin at 6.25 nM, 16.70 (13.39-19.73) at 12.5 nM, 15.23 (11.33-17.74) at 25 nM, 13.16 (10.90-15.78) at 50 nM, and 12.94 (9.95-13.85) at 100 nM. Thus, in Caco-2/TC7 cell monolayers, the FPN mRNA expression was significantly decreased as concentrations of hepcidin were increased in a dose-dependent manner (Figure 5(a); P < 0.001). On the other hand, relative DMT1 mRNA expression in Caco-2/TC7 cell monolayers was 40.51 (11.60-137.7) with no hepcidin, 39.49 (8.42-90.68) with hepcidin at 6.25 nM, 36.49 (16.12-209.8) at 12.5 nM, 31.48 (12.03-91.78) at 25 nM, 24.90 (9.32-161.0) at 50 nM, and 12.94 (9.95-13.85) at 100 nM, showing no significant differences according to hepcidin concentrations (Online Supplemental Figure S1; P = 0.561). Western blot assay was performed to measure expression levels of FPN and DMT1 in Caco-2/TC7 cell monolayers and revealed that FPN expression was increased in accordance with concentrations of hepcidin (Figure 5(b)). There was no correlation between DMT1 expression and hepcidin concentration (Online Supplemental Figure S2).

### 3.7. Immunohistochemical Detection of FPN in Caco-2/TC7 Cell Monolayers

**Figure 6:**
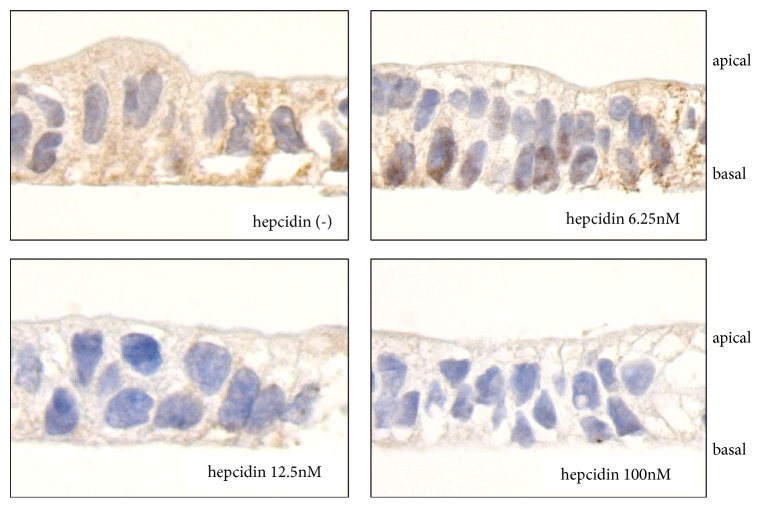
Immunohistochemical detection of FPN in Caco-2/TC7 cell monolayers was cultured with or without hepcidin (0-100 nM). FPN expression in Caco-2/TC7 cell monolayers was downregulated dependently on hepcidin concentration.

We also examined changes in FPN expression in Caco-2/TC7 cell monolayers using an immunohistochemical assay. This assay showed that FPN was strongly expressed in Caco-2/TC7 cell monolayers cultured in the medium without hepcidin, but that heptidin decreased FPN expression in a dose-dependent manner (Figure 6).

## 4. Discussion

Excess iron generates ROS including H_2_0_2_ by the Fenton reaction [4, 19], and oxidative stress is associated with hepatic fibrosis and carcinogenesis [2, 3, 19, 20]. Although hepatic iron overload in CHC patients has been described [21–26], the mechanism of such iron accumulation remains unknown. Possible causes include increased iron absorption from the intestine, increased iron uptake by the liver, and decreased iron release from the liver. To clarify the mechanism of iron overload in the CHC liver, we first examined the dynamics of intestinal iron absorption using the OIAT and found that iron absorption was significantly enhanced in the CHC group compared with the control group. Since iron absorption was enhanced in CHC patients despite the increased Tf sat levels before the OIAT, one possible reason for iron overload could be a breakdown in the negative feedback system that normally suppresses iron absorption in response to systemic iron overload.

Hepcidin was originally isolated from human serum and urine as a peptide with anti-bacterial activity [27–29]. Hepcidin is a hormone produced only in hepatocytes and is involved in iron release by reticuloendothelial macrophages and iron absorption from the intestinal epithelium [8, 30]. Regarding hepcidin expression in CHC patients, the hepcidin mRNA/serum ferritin ratio was shown to be decreased in liver biopsy samples [10, 11] and it was suggested that the decreased DNA-binding ability of C/EBP*α* or HDAC activation by HCV-derived ROS may be responsible for the decreased hepcidin levels in CHC patients [31, 32].

In the present study, we measured serum hepcidin-25 concentrations by LC-MS/MS and found lower concentrations of hepcidin-25 in CHC patients than in the control participants. Though hepcidin is known to inhibit extracellular iron transport by inducing FPN internalization or degradation in Hela cells [7], it remains unclear how hepcidin acts in enterocyte. Our results suggest that there may be tissue-specific differences. We examined mRNA expression levels of molecules related to iron absorption in the duodenum, including FPN and DMT1. Whereas the DMT1 mRNA expression level did not differ between the control and CHC groups, FPN mRNA expression was higher in the CHC group. Similarly, immunohistochemical staining showed increased expression of FPN protein in the duodenum of CHC patients. Thus, it was suggested that hepcidin might regulate FPN expression not only at the protein level but also at the mRNA level in duodenal epithelial cells. We therefore investigated the effects of hepcidin on FPN and DMT1 in a model of duodenal epithelium in which Caco-2/TC7 cells were cultured in transwells as a monolayer for slightly longer than 3 weeks. In this model, DMT1 mRNA expression did not change in response to different concentrations of hepcidin, but FPN mRNA expression decreased by hepcidin in a dose-dependent manner. Based on these findings, one possible mechanism for the iron overload in CHC patients could be associated with enhanced intestinal iron absorption as a result of increased FPN expression in the duodenal mucosa due to decreased serum hepcidin. It should be supported by significant correlation between serum hepcidin-25 and OIAT s-Fe 120 min.

Increased expression of FPN in the duodenum also has been reported in FL-N/35 transgenic mice, a persistent infection model of HCV [31]. In the present study, we found that FPN mRNA expression was suppressed by hepcidin. FPN mRNA expression was reported to be upregulated by Nrf2 in macrophages [5, 33, 34] or by HIF2*α*, which binds to the HIF response elements in enterocytes [35]. However, the association of these molecules with hepcidin was not investigated in our present study and further investigation is needed to clarify the mechanism by which hepcidin suppresses FPN mRNA expression.

In conclusion, our present results showed that the serum hepcidin-25 concentration was decreased in CHC patients, which could enhance iron absorption by increasing the expression of FPN in the duodenum. Results of our* in vitro* assays suggested that hepcidin inhibits mRNA expression of FPN in duodenal epithelial cells. Although currently phlebotomy is used to treat iron overload in patients who have persistent HCV infection, our findings raise the possibility of the development of new drugs based on this mechanism. Furthermore, it was reported that, despite an increasing rate of SVR by direct acting antivirals, hepatic fibrosis is exacerbated in a subset (12%) of patients who achieve SVR [36] and that HCC develops continuously even after achieving SVR [37–39]. Therefore, further investigation of the involvement of abnormal iron absorption in these patients and a preventive strategy based on its underlying mechanism could lead to the establishment of a method to prevent cancer in patients with HCC in the future.

## Figures and Tables

**Table 1:** Baseline characteristics of study participants.

Characteristic	Control group	CHC group	P values
Number of patients	9	24	
Gender, M/F	4/5	11/13	0.9431
Age, y	61 (46-74)	59 (22-73)	0.5709
Body mass index, kg/m2	21.6 (19.0-25.5)	23.3 (16.3-32.5)	0.0688
AST, IU/L	18 (13-32)	56 (28-303)	0.0002
ALT, IU/L	20 (17-30)	71 (35-501)	0.0108
GGT, IU/L	32 (11-64)	52 (10-260)	0.0881
Hb, g/dL	14.8 (11.4-16.2)	13.4 (11.6-16.3)	0.6900
Platelet count, x104/*μ*L	221 (182-265)	176 (57-308)	0.0596
Serum Fe, *μ*g/dL	68 (55-99)	106 (44-234)	0.0038
Serum ferritin, *μ*g/dL	75.0 (25.0-115.0)	184 (12.4-1550)	0.0084
Tf saturation, %	23.8 (19.0-31.8)	41.0 (20.8-63.9)	0.0103
Total cholesterol, mg/dL	201 (140-258)	180 (90-257)	0.1455
Triglyceride, mg/dL	120 (77-235)	118 (64-359)	0.7772

## Data Availability

The data used to support the findings of this study are included within the supplementary information file(s).

## Supplementary Materials

Supplementary Materials Supplemental Figure S1. DMT1 mRNA expression levels in Caco-2/TC7 cell monolayers cultured with various concentrations of hepcidin or without hepcidin were measured by Taqman real-time PCR. Supplemental Figure S2. DMT1 expressions in Caco-2/TC7 cell monolayers cultured with various concentrations of hepcidin or without hepcidin were analyzed by western blot.
