# Supplementary material for: Increased Duodenal Iron Absorption through Upregulation of Ferroportin 1 due to the Decrement in Serum Hepcidin in Patients with Chronic Hepatitis C
Source: Can J Gastroenterol Hepatol. 2018 Aug 14;2018:2154361. doi: 10.1155/2018/2154361 (PMC6112088; doi:10.1155/2018/2154361)

Supplemental Figure S1. DMT1 mRNA expression levels in Caco-2/TC7 cell monolayers cultured with various concentrations of hepcidin or without hepcidin were measured by Taqman real-time PCR. There were no significant differences dependent on hepcidin concentration (P = 0.561; Jonckheere–Terpstra test).

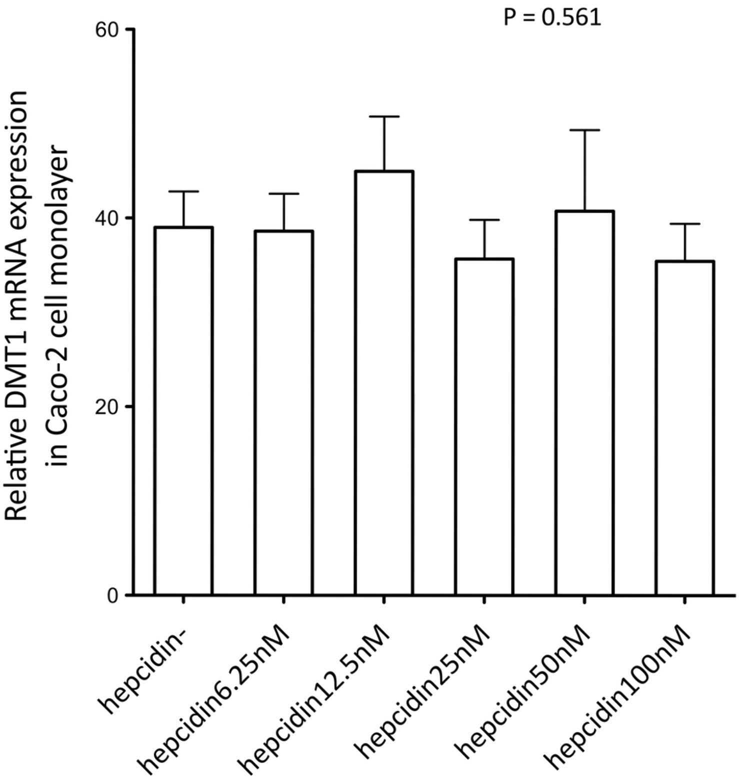

Supplemental Figure S2. DMT1 expression in Caco-2/TC7 cell monolayers cultured with various concentrations of hepcidin or without hepcidin was analyzed by western blot. There were no significant differences dependent on hepcidin concentration.

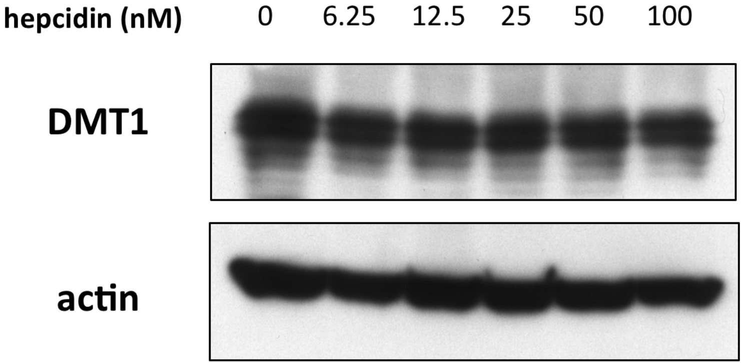

Supplement: Supplementary Materials — Supplemental Figure S1. DMT1 mRNA expression levels in Caco-2/TC7 cell monolayers cultured with various concentrations of hepcidin or without hepcidin were measured by Taqman real-time PCR. Supplemental Figure S2. DMT1 expressions in Caco-2/TC7 cell monolayers cultured with various concentrations of hepcidin or without hepcidin were analyzed by western blot. [file 2154361.f1.pdf]
